# Chronic Rapamycin Prevents Electrophysiological and Morphological Alterations Produced by Conditional Pten Deletion in Mouse Cortex

**DOI:** 10.3390/cells14020079

**Published:** 2025-01-08

**Authors:** Jason S. Hauptman, Joseph Antonios, Gary W. Mathern, Michael S. Levine, Carlos Cepeda

**Affiliations:** 1 IDDRC, Jane and Terry Semel Institute for Neuroscience and Human Behavior, David Geffen School of Medicine, University of California Los Angeles, Los Angeles, CA 90095, USA; jhauptman1@phoenixchildrens.com (J.S.H.); joseph.antonios@yale.edu (J.A.); gmathern@ucla.edu (G.W.M.);; 2 Department of Neurosurgery, David Geffen School of Medicine, University of California Los Angeles, Los Angeles, CA 90095, USA

**Keywords:** mTOR, rapamycin, electrophysiology, cerebral cortex

## Abstract

Abnormalities in the mammalian target of the rapamycin (mTOR) pathway have been implicated in numerous developmental brain disorders. While the molecular and histological abnormalities have been described, less is known about alterations in membrane and synaptic excitability with chronic changes in the mTOR pathway. In the present study, we used a conditional mouse model with a deletion of the phosphatase and tensin homologue (Pten^-/-^, a negative regulator of mTOR) from cortical pyramidal neurons (CPNs). Whole-cell patch clamp recordings in ex vivo slices examined the intrinsic and synaptic membrane properties of layer II/III CPNs in normal mice treated with rapamycin for four weeks, and Pten^-/-^ mice with and without chronic treatment with rapamycin. Compared with control mice, CPNs from Pten^-/-^ mice demonstrated increased membrane capacitance and time constant in association with increased neuronal somatic size, reduced neuronal firing, and decreased frequency of spontaneous and miniature inhibitory postsynaptic currents, consistent with decreased pre-synaptic GABA release. Rapamycin treatment for four weeks prevented these changes in Pten^-/-^ mice. CPNs from normal mice chronically treated with rapamycin, compared with CPNs from naïve mice, showed reduced capacitance and time constant, increased input resistance, and changes in inhibitory synaptic inputs, consistent with increased pre-synaptic GABA release. These results support the concept that Pten deletion results in significant changes in inhibitory inputs onto CPNs, and these alterations can be prevented with chronic rapamycin treatment. In addition, normal mice treated with rapamycin also display altered membrane and synaptic properties. These findings have potential implications for the treatment of neurological disorders associated with mTOR pathway dysfunction, such as epilepsy and autism.

## 1. Introduction

The mechanistic target of rapamycin (mTOR), a ubiquitous ~289 kDa serine/threonine kinase, has been recognized as a crucial regulator of diverse cellular processes and an integral modulator of cellular homeostasis [1,2,3,4,5]. In addition to cell growth and cell cycle progression, the mTOR pathway regulates mitochondrial biogenesis, lipid synthesis, autophagy, ribosome biogenesis, and RNA translation [6]. In the central nervous system, dysregulation of the mTOR pathway has been implicated in developmental abnormalities including tuberous sclerosis complex (TSC), focal cortical dysplasia, brain tumors, hemimegalencephaly, and autism spectrum disorders [7,8,9].

The activation of mTOR results from the binding of extracellular ligands to receptor tyrosine kinases, which in turn stimulate PI3K/Akt signaling—for reviews, see [1,10]. Upstream activation of mTOR is negatively regulated by the phosphatase and tensin homologue (Pten), a major antagonist of PI3K/Akt-dependent signaling [11]. Pten mutations also have been implicated in tumorigenesis, malformations of cortical development, and autism [12,13]. While the molecular biology of the mTOR pathway has been described in detail, less is known about the role of Pten in neuronal excitability. In animal models, it has been shown that the deletion of Pten in the brain causes cell overgrowth and seizures, among other outcomes [14,15,16,17,18]. Notably, inhibition of the mTOR pathway suppresses seizures and neuronal hypertrophy [19,20,21]. Furthermore, homozygous mice with a conditional deletion of Pten specifically from excitatory forebrain neurons display enlargement of the forebrain and die shortly after birth [22]. The majority of previous studies looking at probable causes of seizures have focused on the hippocampus, which displays morphological and electrophysiological alterations after Pten deletion [23,24,25,26,27,28]. These studies agree that Pten loss leads to altered membrane properties, increased excitatory synaptic transmission, hyperexcitability, and hypertrophy. There are fewer reports examining the consequences of Pten deletion from cortical pyramidal neurons (CPNs), as well as on its effects on inhibitory synaptic transmission [29,30].

The present study was designed to determine the role of the Pten in the modulation of CPN somatic morphology and electrophysiology in a mouse model with a conditional deletion of Pten from CPNs, with particular emphasis on changes in GABAergic neurotransmission, a subject rarely examined in previous studies. We also examined the effects of rapamycin, which allosterically inhibits mTORC1 [6], using a combination of electrophysiological, pharmacological and genetic strategies to manipulate PI3K-Akt signaling and subsequent mTOR activation. Our findings demonstrate that the mTOR pathway plays a central role in the modulation of the basic membrane properties, intrinsic excitability, GABA synaptic activity, and morphology of CPNs. Further, abnormalities produced by Pten deletion can be prevented with chronic rapamycin treatment.

## 2. Materials and Methods

### 2.1. Experimental Animals and Breeding Strategies

Wild-type (WT) C57BL6/J mice were purchased from Jackson Laboratories (Bar Harbor, ME, USA) and used to form C57BL6/J breeding colonies in our facilities at the University of California Los Angeles (UCLA, Los Angeles, CA, USA). Mice with floxed Pten alleles (Pten^flox/flox^) and αCaMKII-Cre transgenic mice on a C57BL6 congenic background were also obtained from The Jackson Laboratory. The breeding of Pten^flox/flox^ mice with Pten^wt/wt^ αCaMKII-Cre^+/Tg^ mice resulted in litters of Pten^flox/wt^ αCaMKII-Cre^+/Tg^ mice. These animals were then backcrossed to the Pten^flox/flox^ mice to generate Pten^flox/flox^ αCaMKII-Cre^+/Tg^ mice (denoted as Pten^-/-^). Pten^flox/flox^ αCaMKII-Cre^-/Tg^ littermates were used as controls in all studies involving mutants. Loss of Pten expression from the excitatory CPNs of Pten^-/-^ mice starts around P30, and by P60 about 50% of neurons lack Pten expression [31].

All mice were used for electrophysiological recordings at around postnatal day (P) 60. Experimental procedures were performed in accordance with the United States Public Health Service Guide for Care and Use of Laboratory Animals and were approved by the Institutional Animal Care and Use Committee at UCLA. Every effort was made to minimize pain and discomfort, as well as the number of animals utilized.

### 2.2. Brain Slice Preparation and Whole-Cell Patch Clamp Recordings

Detailed procedures have been previously published [32]. Briefly, mice were deeply anesthetized with isoflurane. The brains were dissected and immediately placed in oxygenated ice-cold low-calcium artificial cerebrospinal fluid (ACSF) containing (in mM); NaCl, 130; KCl, 3; NaH_2_PO_4_, 1.25; NaHCO_3_, 26; MgCl_2_, 5; CaCl_2_, 1; and glucose, 10. Coronal slices of the frontal cortex (350 µm) were cut and transferred to an incubating chamber containing ACSF (with 2 mM CaCl_2_ and 2 mM MgCl_2_) oxygenated with 95% O_2_-5% CO_2_ (pH 7.2–7.4, 290–310 mOsm, 25 ± 2 °C). After 1 h of incubation, slices were transferred to the recording chamber.

Whole-cell patch clamp recordings were obtained from layer II/III CPNs identified by somatic shape and size using infrared videomicroscopy and differential interference contrast (IR-DIC) optics. For voltage clamp recordings, patch electrodes (3–4.5 MΩ) were filled with (in mM) Cs-methanesulfonate 130, CsCl 10, NaCl 4, MgCl_2_ 1, MgATP 5, EGTA 5, HEPES 10, GTP 0.5, phosphocreatine 10, and leupeptin 0.1 (pH 7.25–7.3, osmolality 280–290 mOsm). For current clamp recordings, electrodes were filled with (in mM) K-gluconate 140, HEPES 10, MgCl_2_ 2, CaCl_2_ 0.1, EGTA 1.1, and K_2_ATP 2. Cells were patched, (Multipatch 700B, Molecular Devices, Sunnyvale, CA, USA) and basic membrane properties were recorded using pClamp (v. 10.2) software (Axon Instruments, Burlingame, CA, USA). Series resistance and cell capacitance were compensated as necessary. Currents were filtered at 1 kHz and digitized at 100–200 µsec.

Spontaneous excitatory and inhibitory postsynaptic currents (sEPSCs and sIPSCs, respectively) were recorded in voltage clamp mode over a three-minute period. When pharmacological agents were used, recording was begun approximately six minutes after the application of the drug. For sEPSCs, the neuronal membranes were voltage-clamped at −70 mV and 10 µM bicuculline was applied to block GABA_A_ receptor-mediated events. For sIPSCs, the cell membranes were depolarized from −70 mV to +10 mV and 10 µM 2,3-dihydroxy-6-nitro-7-sulfamoyl-benzo[f]quinoxaline-2,3-dione (NBQX), an AMPA receptor antagonist, and 100 µM (2R)-amino 5-phosphonopentanoate (APV), an NMDA receptor antagonist, were applied to block ionotropic glutamate receptors. For miniature (m)EPSCs and mIPSCs, 1 µM tetrodotoxin (TTX) was bath applied to block voltage-gated Na^+^ conductances. Bicuculline, TTX, NBQX, and APV were obtained from Tocris Pharmaceuticals (Minneapolis, MN, USA). Data were subsequently analyzed off-line using the automatic event detection program in MiniAnalysis (Synaptosoft, Fort Lee, NJ, USA) and then reviewed manually for accuracy. The root mean square background noise was 2–3 pA. The amplitude threshold was 5 pA for sEPSCs and 10 pA for sIPSCs. Amplitude–frequency, cumulative amplitude, and cumulative inter-event interval distributions were constructed to evaluate differences in events at each amplitude and interval. To analyze kinetics, events were grouped using amplitude criteria (5–50 pA for sEPSCs and 10–100 pA for sIPSCs), normalized and averaged. The decays of averaged events were fit to either second- or third-order exponentials (depending on best fit).

Synaptic currents were evoked with a monopolar glass stimulating electrode (ACSF-filled patch-pipette, impedance 1–1.2 MΩ) placed approximately 200 µm from the recorded cell. For evoked (e)EPSCs, stimulation electrodes were placed in layer I to activate thalamocortical and corticocortical glutamatergic afferents. Neurons were voltage-clamped at −70 mV and picrotoxin (PTX, 10 µM, a GABA_A_ receptor antagonist) (Sigma Aldrich, St. Louis, MO, USA) was applied. For eIPSCs, the monopolar electrode was positioned within layer II/III to maximally activate afferents of local GABAergic interneurons. The cell membranes were depolarized to a holding potential of +10 mV for five seconds prior to stimulation and for one second following stimulation. Then, 10 µM NBQX and 100 µM APV were applied. Three consecutive trials of test stimuli (0.5 msec duration) at increasing intensities (0.01–0.10 mA) were applied every 20 s and responses were averaged. Using 50% of the maximal intensity, the probability of neurotransmitter release was estimated using paired-pulse stimulation (25–400 msec inter-pulse intervals). Paired-pulse facilitation (PPF) was defined as ratios of pulse2/pulse1 of 1.1 or greater and paired-pulse depression (PPD) was defined as similar ratios of 0.9 or less.

For measurements of intrinsic cellular excitability, a K-gluconate internal solution was used. Initially, cells were patched in voltage clamp mode and passive membrane properties were recorded at V_hold_ = −70 mV. In current clamp mode, a series of current injections (steps from −250 pA to +200 pA in 50 pA increments, 100 msec duration) were applied. Resting membrane potential (RMP), action potential (AP) threshold and current–voltage (IV) relationships were calculated. A similar protocol, but using one-second-duration current steps, was used to calculate the number of APs generated at each step.

### 2.3. Chronic Rapamycin Administration Protocol

Rapamycin was obtained from LC Laboratories (Boston, MA, USA). For rapamycin injections, 25 mg of rapamycin were initially dissolved in 1 mL of ethanol and kept frozen at −25 °C. On the days of injection, WT C57BL6/J male and female mice received intraperitoneal (IP) injections of 10 mg/kg rapamycin dissolved in 4% ethanol, 5% PEG, and 5% Tween 80 every other day for four weeks starting at P30. Pten^-/-^ animals that underwent rapamycin treatment also received IP injections using the same protocol.

### 2.4. Morphological Analysis

Somatic cross-sectional areas were calculated using ImageJ (v. 1.42) software (USA) by an experimenter blind to the genotype from photomicrographs of IR-DIC images obtained at the time of recording. All cortical neurons whose borders were clearly demarcated from the surrounding neuropil were traced for area measurement.

### 2.5. Data Analysis

Electrophysiological data were collected and analyzed off-line using pClamp 10.2 and MiniAnalysis programs. Differences between group means for all measures were compared using Student’s *t*-tests (for two group comparisons) and appropriate one- or two-way ANOVAs followed by Bonferroni *t*-tests (multiple group comparisons) using SigmaStat software (SPSS, v. 3.5, Chicago, IL, USA). Differences were considered statistically significant when *p* < 0.05. All numerical values for data are represented in the text, graphs, and figures as the mean ± standard error of the mean (SEM).

## 3. Results

### 3.1. Alterations in CPN Morphology and Electrophysiology Following Pten Deletion

Starting at 4 weeks of age, exponentially more CPNs express conditional Pten loss to the point that, at 8 weeks of age, approximately half of all CPNs have undergone deletion [31]. Consistent with previous reports, following Pten deletion, we found that by P60, these animals demonstrated macrocephaly, enlarged brains, and age-dependent spontaneous seizures [21,33,34]. Loss of Pten in CPNs resulted in changes to basic membrane properties. Cells recorded from the Pten^-/-^ neocortex displayed increases in membrane capacitance (145 ± 5 pF in WT vs. 189 ± 8 pF in Pten^-/-^, *p* < 0.001, Student’s *t*-test), decay time constant (3.5 ± 0.2 ms in WT vs. 4.3 ± 0.2 ms in Pten^-/-^, *p* < 0.01), and a trend towards a decrease in input resistance (116 ± 7 MΩ in WT vs. 98 ± 9 MΩ in Pten^-/-^, *p* = 0.12), suggesting that Pten loss in CPNs results in larger cells.

**Figure 1:**
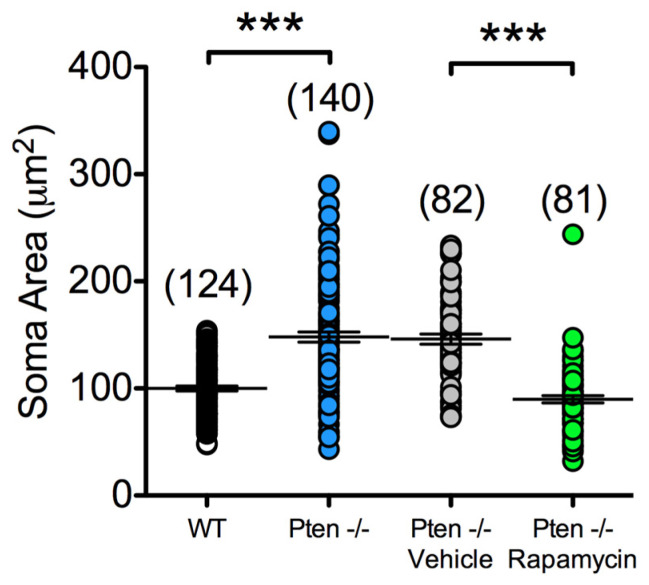
Pten- and mTOR-dependent changes in somatic area. The left two distributions are from WTs and Pten^-/-^ mice and the right two distributions are from rapamycin- and vehicle-treated Pten^-/-^ mice. The difference among groups was statistically significant (F_(3,423)_ = 64.80, *p* < 0.001, One-way ANOVA). Bonferroni’s multiple comparisons test indicated significant differences between WT and Pten^-/-^ mice (*p* < 0.001) and also between Pten^-/-^ Vehicle and Pten^-/-^ Rapamycin (***, *p* < 0.001).

To verify the increase in size, we examined the cross-sectional area of visualized neurons from mice in slices using IR-DIC microscopy. Compared to WT, CPNs from Pten^-/-^ mice displayed larger cross-sectional somatic areas (100 ± 2 µm^2^ in WT vs. 148 ± 5 µm^2^ in Pten^-/-^, *p* < 0.001) (Figure 1, left two distributions). While all CPNs had cross-sectional areas of <200 µm^2^ in WT mice, many CPNs from Pten^-/-^ mice had areas >200 µm^2^, with some neurons having areas approaching 300 µm^2^ or more. However, there was marked variability in cell areas within the Pten^-/-^ neocortex. This likely reflects the fact that approximately 50% of CPNs are expected to undergo Pten deletion by P60 [31].

**Figure 2:**
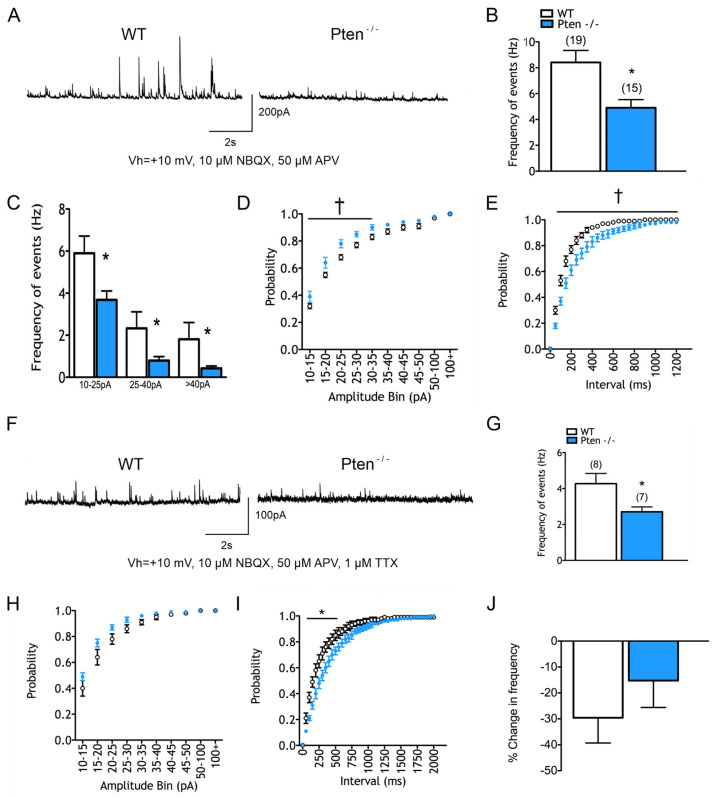
Changes in inhibition following Pten deletion from neocortex. (**A**) Sample sIPSC traces. Note the decrease in sIPSCs in cells from the Pten^-/-^ cortex. (**B**) Mean frequency of sIPSCs was significantly reduced in cells from the Pten^-/-^ neocortex. (**C**) Changes in sIPSCs after binning by amplitude. Decreases occurred in all bins. (**D**) sIPSC cumulative amplitude plot demonstrating significantly reduced amplitudes. (**E**) sIPSC inter-event interval histogram showing that events were significantly further apart. (**F**) Sample mIPSC traces. (**G**) Mean frequency of mIPSCs was significantly reduced in cells from the Pten^-/-^ neocortex. (**H**) mIPSC cumulative amplitude plot demonstrating a slight but not statistically significant trend for reduced amplitudes. (**I**) mIPSC inter-event interval histogram showing that events were significantly further apart. (**J**) Percent changes in mean IPSC frequency following the addition of TTX were not significantly different, although there was a trend of decrease in cells from the Pten^-/-^ cortex.

sEPSCs were recorded and found to be unchanged between groups. Notably, there was a significant reduction in the mean total frequency of sIPSCs in cells recorded from Pten^-/-^ CPNs (4.9 *±* 0.6 Hz in Pten^-/-^ vs. 8.4 *±* 0.9 Hz in WT, *p* = 0.006) (Figure 2A,B). This decrease was present in all bins of the amplitude–frequency distribution (F_(1,32)_ = 8.787 for genotype effect, *p* = 0.0057) (Figure 2C). The cumulative amplitude probability plot showed that sIPSCs had smaller amplitudes in cells from the Pten^-/-^ neocortex (F_(1,32)_ = 5.089 for genotype effect, *p* = 0.031) (Figure 2D). The cumulative inter-event interval probability histogram showed a reduced number of events across all intervals (F_(24,768)_ = 8.204 for interaction effect, F_(1,32)_ = 10.64 for genotype effect, *p* < 0.001 and *p* = 0026 respectively) (Figure 2E). There were no significant changes in the kinetics of average GABAergic events.

To determine whether the changes in sIPSC frequencies were due to pre- or postsynaptic alterations, mIPSCs were isolated (Figure 2F). There was a decrease in the mean mIPSC frequency in the Pten^-/-^ neocortex (2.7 *±* 0.3 Hz in Pten^-/-^ vs. 4.3 *±* 0.6 Hz in WT, *p* = 0.03) (Figure 2G), paralleling the observed reduction in sIPSC frequency. Even though mean mIPSC amplitudes were decreased in Pten^-/-^ animals, the percent change in frequency before and after TTX was not different from that of WTs (Figure 2J). This suggests that the reduction in GABAergic activity is independent of AP-mediated neurotransmitter release. The cumulative amplitude probability histogram indicated a trend of smaller mIPSCs amplitudes in the Pten^-/-^ neocortex, particularly in the 20–25 and 25–30 pA bins, but statistical significance was not reached (F_(1,13)_ = 2.873 for genotype effect, *p* = 0.114) (Figure 2H). The inter-event interval probability distributions showed that fewer mIPSCs occurred in close temporal proximity to each other in neurons recorded from Pten^-/-^ mice (F_(40,520)_ = 6.696 for interaction and F_(1,13)_ = 162.22 for genotype effect, *p* < 0.001 and *p* = 0.0292 respectively) (Figure 2I). No significant changes occurred in mIPSC kinetics. Together, these data suggest that the decreased frequency of spontaneous GABAergic currents may be due to a reduction in presynaptic neurotransmitter release.

**Figure 3:**
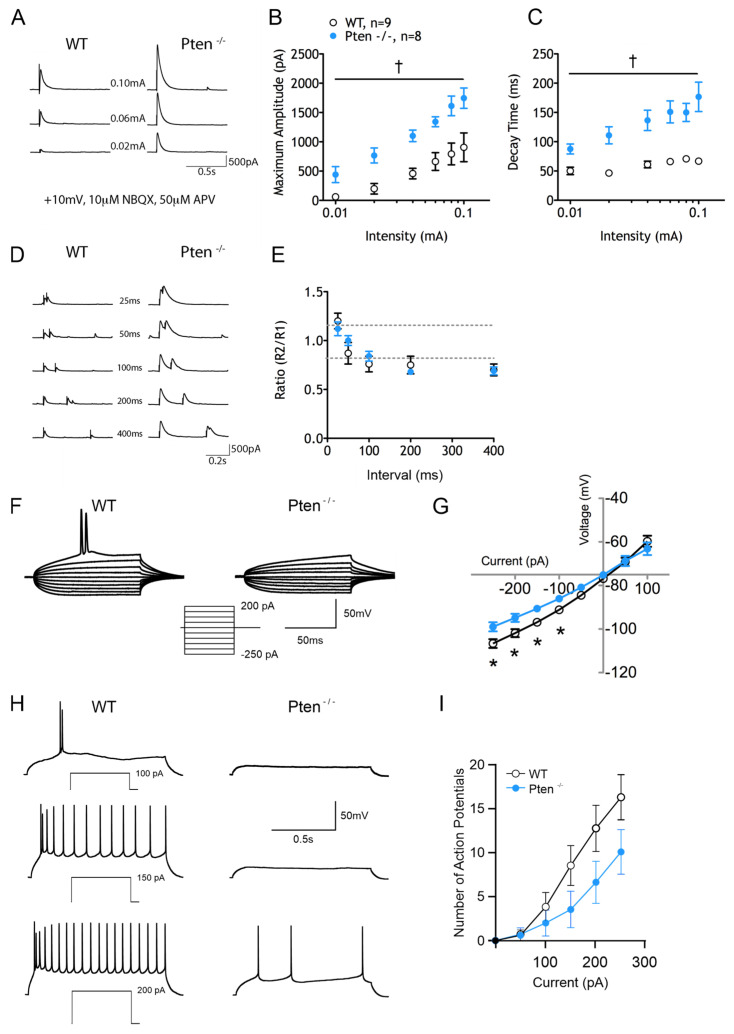
Cells from the Pten^-/-^ neocortex exhibit changes in inhibition and membrane excitability. (**A**) Sample eIPSC traces. (**B**) Significantly increased mean eIPSC amplitudes in cells from Pten^-/-^ neocortex. (**C**) Significantly longer eIPSC decay times in cells from the Pten^-/-^ neocortex. (**D**,**E**) Graphs showing no changes in paired-pulse ratios. (**F**) Examples of 100 ms I–V traces. (**G**) Significant changes in rectification in the hyperpolarizing direction in cells from the Pten^-/-^ neocortex. (**H**) Examples of 1 s current injections inducing action potentials. (**I**) Fewer APs were generated in cells from the Pten^-/-^ neocortex in response to depolarizing current injection. However, due to neuronal variability, the difference did not reach statistical significance.

Pten deletion also altered the eIPSC input–output function of CPNs (Figure 3A). Surprisingly, the maximum amplitudes of eIPSCs were larger in the Pten^-/-^ neocortex (F_(1,15)_ = 13.99 for genotype effect, *p* = 0.002) (Figure 3B), and there was an increase in eIPSC decay times (F_(1,15)_ = 32.38 for genotype effect, *p* < 0.001) (Figure 3C). To adjust for the confounding increase in capacitance, eIPSCs were normalized by capacitance to analyze the evoked GABAergic current density. Even with the increase in capacitance in CPNs recorded from the Pten^-/-^ neocortex, the significant increase in current density in eIPSCs persisted (F_(1,15)_ = 4.371 for genotype effect, *p* = 0.05). Post hoc tests revealed statistically significant differences at 0.01 to 0.06 pA current intensities. To determine if the increase in eIPSC amplitudes was due to a change in P_r_, paired-pulse ratios were examined (Figure 3D). Neurons from both the Pten^-/-^ and WT neocortex displayed paired-pulse facilitation at the 25 msec interval and paired-pulse depression (PPD) at intervals of 100, 200, and 400 msec. There were no differences in paired-pulse ratios between Pten^-/-^ and WT animals at any inter-stimulus interval (Figure 3E), suggesting that any changes in eIPSC amplitudes were not due to changes in release probability (P_r_).

In general, membrane excitability was decreased in CPNs recorded from the Pten^-/-^ neocortex (Figure 3F). Although neurons in the two groups had similar RMPs, membrane potentials at the AP threshold, ΔV, and AP kinetics, there was an effect of Pten deletion on the IV curve. While the overall genotype effect was not statistically significant (F_(1,23)_ = 3.05, *p* = 0.094), the Bonferroni post hoc test revealed significant differences at hyperpolarized potentials (Figure 3G). This effect resulted in increased inward rectification.

Cells from the WT and Pten^-/-^ neocortex differed with respect to the input–output relationship, as shown from the 1 s current step protocol (Figure 3H). Although neurons from the Pten^-/-^ neocortex usually generated fewer APs at each current step, the difference did not reach statistical significance (F_(1,22)_ = 2.273 for genotype effect, *p* = 0.145). The Bonferroni post hoc test indicated a trend for reduced firing with 200–250 pA current injections (*p* = 0.0975 and *p* = 0.0993 respectively) (Figure 3I).

### 3.2. Rapamycin Prevents the Effects of Pten^-/-^ Deletion

These experiments examined the effects of treating Pten^-/-^ mice with rapamycin. Pten^-/-^ mice received either rapamycin (10 mg/kg) or vehicle IP every other day for 4 weeks starting at P30. At about P60, animals were used for electrophysiological recordings to determine if rapamycin prevented the effects of Pten deletion. When Cs-methanesulfonate was used as the internal solution, neurons from rapamycin-treated Pten^-/-^ mice did not display changes in capacitance (175 ± 9 pF in Pten^-/-^ vehicle vs. 139 ± 8 pF in Pten^-/-^ rapamycin, *p* < 0.01, Student’s *t*-test) or input resistance (108 ± 11 MΩ in Pten^-/-^ vehicle vs. 159 ± 19 MΩ in Pten^-/-^ rapamycin, *p* < 0.05), but they displayed a strong trend of having a reduced time constant (4.4 ± 0.3 ms in Pten^-/-^ vehicle vs. 2.8 ± 0.2 ms in Pten^-/-^ rapamycin. In agreement, compared to the vehicle, CPNs from rapamycin-treated Pten^-/-^ mutants displayed significantly smaller somatic cross-sectional areas (146 ± 5 µm^2^ in vehicle vs. 90 ± 3 µm^2^ in rapamycin, *p* < 0.001) (Figure 1, right two distributions). The mean cross-sectional area was similar to values obtained from untreated WT mice (100 ± 2 µm^2^), and the distributions were similar. Thus, rapamycin treatment prevented the hypertrophy caused by Pten deletion.

**Figure 4:**
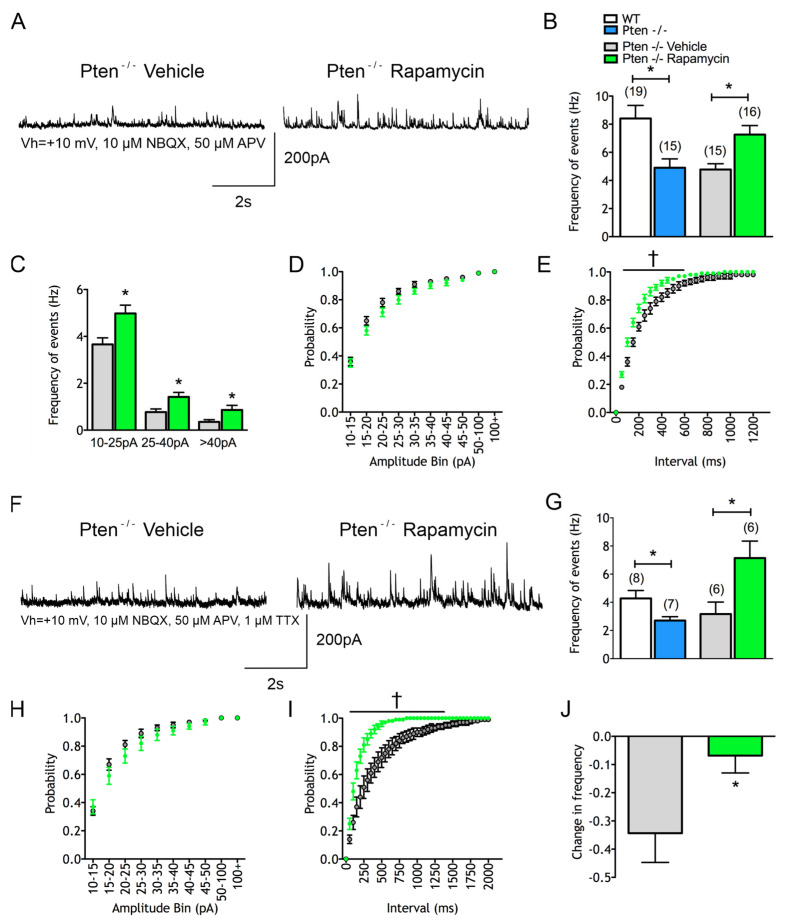
Rapamycin prevents changes in inhibitory synaptic activity within the Pten^-/-^ neocortex. (**A**) Sample traces of sIPSCs. (**B**) Rapamycin significantly increases mean sIPSC frequency. (**C**,**D**) Rapamycin significantly increases sIPSC frequencies across amplitude bins and sIPSC cumulative amplitude plot demonstrated significantly increased amplitudes. (**E**) Rapamycin significantly increased the temporal proximity of sIPSC events. (**F**) Sample traces of mIPSCs. (**G**–**I**) Increases in mIPSCs caused by rapamycin follow the pattern of changes in sIPSCs. (**J**) The percent change in frequency of IPSCs following TTX was significantly reduced following rapamycin, suggesting that the majority of sIPSC events are independent of action potentials.

Following rapamycin treatment, CPNs from the Pten^-/-^ neocortex displayed increases in sIPSCs compared to neurons from the Pten^-/-^ neocortex from mice that did not receive rapamycin (4.8 ± 0.4 Hz in vehicle vs. 7.3 ± 0.7 Hz in rapamycin, *p* = 0.003) (Figure 4A,B). This increase occurred across all amplitude bins (F_(1,29)_ = 10.23 for genotype effect, *p* = 0.0033) (Figure 4C). The cumulative amplitude probability histogram demonstrated a subtle but not statistically significant increase in sIPSCs across amplitudes following chronic rapamycin treatment (F_(1,29)_ = 2.019) for treatment effect, *p* = 0.166) (Figure 4D). The inter-event interval probability histogram showed that rapamycin increased the temporal proximity of GABAergic events (F_(1,29)_ = 7.01 for treatment effect and F_(24,696)_ = 6.753 for interaction effect, *p* = 0.013 and *p* < 0.001 respectively) (Figure 4E). Rapamycin also resulted in faster decay time kinetics of averaged sIPSCs (18 ± 1.4 msec in vehicle vs. 13 ± 0.7 msec in rapamycin, *p* = 0.003). There also was a trend toward shorter half-amplitude durations (18 ± 0.5 msec in vehicle vs. 17 ± 0.4 msec in rapamycin, *p* = 0.07) and rise times (1.4 ± 0.1 msec in vehicle vs. 1.2 ± 0.1 msec in rapamycin, *p* = 0.09). Together, these results suggest that there may be a Pten-independent, mTOR-dependent effect on kinetics that only occurs when mTOR is unregulated.

Rapamycin treatment increased mIPSC frequency in the Pten^-/-^ neocortex (3.0 ± 0.9 Hz in vehicle vs. 7.1 ± 1.2 Hz in rapamycin, *p* = 0.003) (Figure 4F,G). There was less of a change in IPSC frequency after TTX in the rapamycin-treated group (−34.3 ± 10% in vehicle vs. −6.8 ± 6% in rapamycin, *p* = 0.05) (Figure 4J), suggesting that the rapamycin-induced increase in GABAergic events was AP-independent. There was a subtle but not statistically significant increase in the proportion of mIPSCs that had larger amplitudes (F_(1,10)_ = 2.844 for treatment effect, *p* = 0.1226) (Figure 4H), and rapamycin resulted in a greater temporal proximity of mIPSC events (F_(1,10)_ = 10.83 for treatment effect and F_(40,400)_ = 9.823 for interaction effect, *p* = 0.008 and *p* < 0.001 respectively) (Figure 4I). Similarly to the findings from the untreated Pten^-/-^ mutants, compared to those from the WT littermates, the effect on IPSCs appears to be contributed primarily by a presynaptic change.

**Figure 5:**
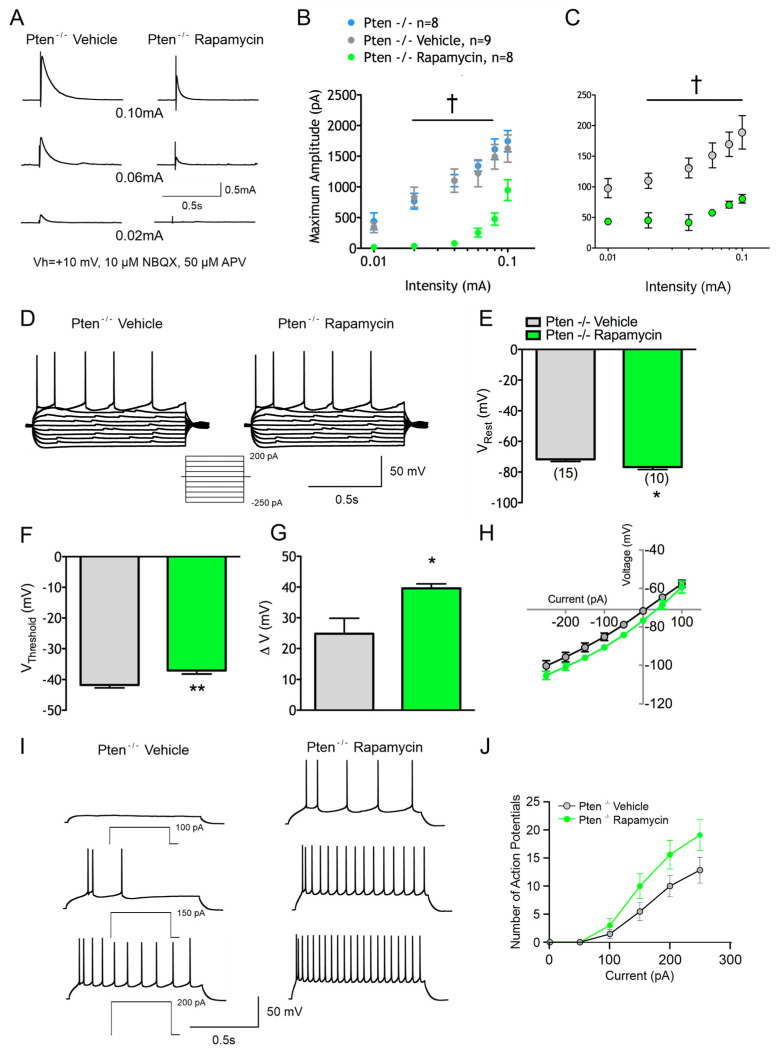
Rapamycin prevents changes in the synaptic responses and membrane excitability of the Pten^-/-^ neocortex. (**A**) Sample eIPSC traces. (**B**) Rapamycin significantly reduced mean eIPSC amplitudes. (**C**) Rapamycin significantly shortens mean eIPSC decay times. (**D**) Sample 1 s I–V traces recorded from vehicle- and rapamycin-treated neurons. (**E**) Rapamycin resulted in a significantly more hyperpolarized RMP. (**F**) Rapamycin resulted in a significantly more depolarized threshold for the action potential. (**G**) Rapamycin resulted in a significantly increased voltage change to reach the threshold for APs from the RMP. (**H**) Rapamycin significantly shifted the I–V curve in the hyperpolarizing direction. (**I**,**J**) Rapamycin increased the number of APs generated by the injection of depolarizing current.

Rapamycin also reduced eIPSC amplitudes (F_(2,22)_ = 12.75 for treatment effect, *p* = 0.002) at all but the highest intensity of stimulation (Figure 5A,B). The Pten^-/-^ effect on eIPSC decay times was prevented by rapamycin (F_(1,15)_ = 17.56 for treatment effect, *p* = 0.008) (Figure 5C). Again, paired-pulse ratios were unchanged, suggesting that rapamycin’s effect was not mediated through changes in Pr. Rapamycin treatment also changed membrane excitability (Figure 5D).

The RMP was more hyperpolarized in rapamycin-treated Pten animals (−71.7 ± 1.4 mV in vehicle vs. −76.7 ± 1.7 mV in rapamycin, *p* = 0.03) (Figure 5E), and there was a more depolarized threshold for APs (−41.7 ± 0.9 mV in vehicle vs. −37.13 ± 1.1 mV in rapamycin, *p* < 0.01) (Figure 5F), leading to a greater ΔV (24.8 ± 5.0 mV in vehicle vs. 39.6 ± 1.4 mV in rapamycin, *p* = 0.02) (Figure 5G). These findings suggest that a greater depolarizing current is necessary to cause cells from the rapamycin-treated Pten^-/-^ neocortex to fire APs. Rapamycin treatment altered the I–V relationship, shifting the curve towards more hyperpolarized potentials with positive current injection (F_(1,21)_ = 4.662 for treatment effect, *p* = 0.0426) (Figure 5H).

Pten^-/-^ animals showed a small reduction in the number of APs generated by a given depolarizing current injection compared to WT animals. Rapamycin treatment prevented this effect, with cells from the treated Pten^-/-^ neocortex displaying a higher frequency of APs with depolarizing current steps (F_(1,21)_ = 3.08 for treatment effect and F_(5,104)_ = 2.68 for interaction effect, *p* = 0.0935 and *p* = 0.0254 respectively) (Figure 5I,J).

### 3.3. In Normal Cortex, Chronic Rapamycin Alters Basic Membrane Properties, Reduces Intrinsic Excitability, and Increases Spontaneous GABA Release

To examine the effects of mTOR inactivation in intact brains, WT C57BL6/J mice received rapamycin (10 mg/kg, IP) or the vehicle every other day for four weeks starting at P30. Layer II/III CPNs from mice treated with rapamycin exhibited a reduction in membrane capacitance (*p* = 0.001) and time constant (*p* = 0.01), and an increase in input resistance (*p* = 0.03) when measured in voltage clamp mode using Cs-methanesulfonate as the internal solution. In contrast, using K-gluconate, the same trends were observed but the differences did not reach statistical significance. This indicates that the blockade of inwardly rectifying K^+^ channels with Cs^+^ is necessary to reveal alterations in the basic membrane properties of CPNs from animals treated with rapamycin.

**Figure 6:**
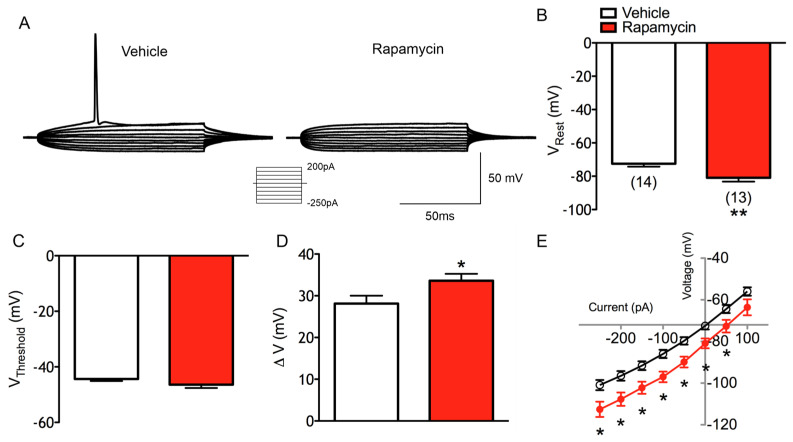
Changes in WT CPN membrane excitability after chronic rapamycin. (**A**) Example of I–V traces in response to 100 msec current pulses. (**B**) Chronic rapamycin induced significantly greater hyperpolarization of the RMP. (**C**) Graph showing that threshold for AP generation was not increased by chronic rapamycin. (**D**) Graph showing that the voltage change from V_rest_ to V_threshold_ was significantly increased by chronic rapamycin. (**E**) I–V relationship demonstrating that membrane potentials were significantly more hyperpolarized after chronic rapamycin treatment at all current steps.

Excitability was examined in current clamp mode using the K-gluconate-based internal solution. A reduction in membrane excitability occurred (Figure 6A). Compared with the vehicle, normal CPNs recorded from chronic rapamycin-treated mice were more hyperpolarized at rest (−72.0 ± 1.7 mV in vehicle vs. −80.9 ± 2.4 mV in rapamycin, *p* = 0.004) (Figure 6B). Chronic rapamycin did not result in a change in the threshold potential to elicit APs (−44.38 ± 0.7 mV in vehicle vs. −46.41 ± 1.2 mV in rapamycin) (Figure 6C). Because of the more hyperpolarized RMP in rapamycin-treated neurons, the ΔV was increased (28.12 ± 1.9 mV in vehicle vs. 33.62 ± 1.6 mV in rapamycin, *p* = 0.04) (Figure 6D). Membrane potentials were more hyperpolarized in the rapamycin group at all current steps (F(1,26) = 12.43 for treatment effect, *p* = 0.0016) (Figure 6E). In the IV plots, chronic rapamycin did not alter the rectification (i.e., slope) in either direction. Instead, there was a downward (i.e., hyperpolarizing) shift in the I–V curve at all but the most depolarized membrane potential.

**Figure 7:**
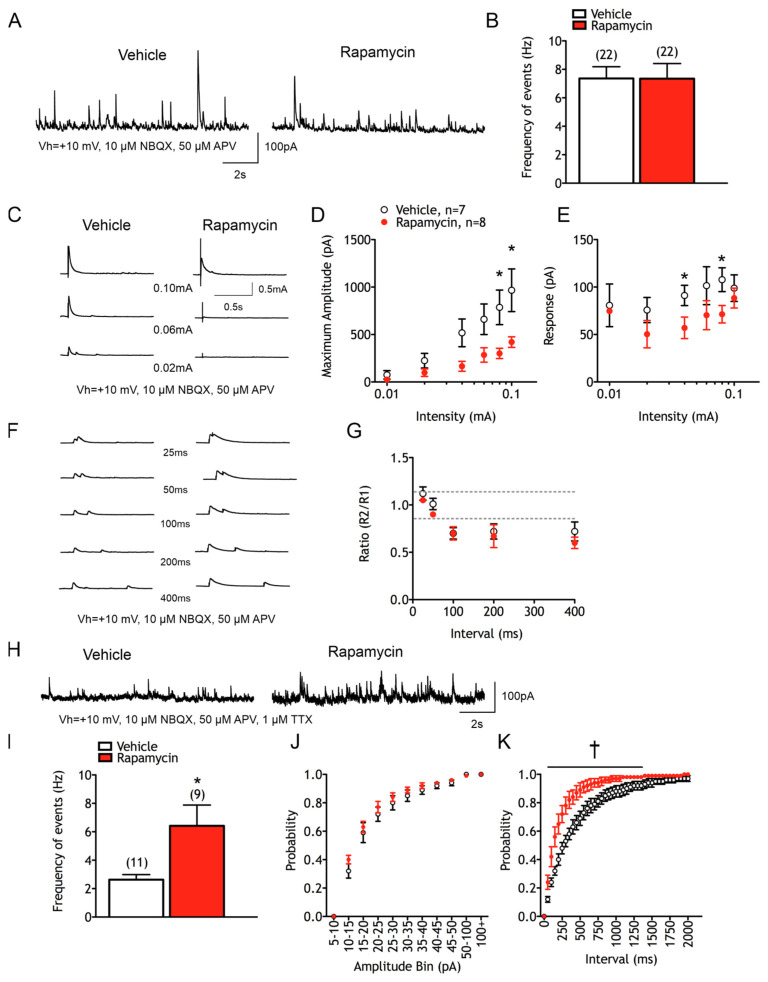
Chronic rapamycin treatment alters synaptic inhibition in an intact neocortex. (**A**) Sample sIPSC traces. (**B**) Graph showing that chronic rapamycin treatment did not alter mean sIPSC frequency. (**C**) Sample traces of eIPSCs. (**D**) Chronic rapamycin significantly reduced mean eIPSC amplitudes. (**E**) Chronic rapamycin treatment significantly reduced eIPSC decay times (significant omnibus effect). (**F**) Sample paired-pulse traces of eIPSCs. (**G**) Graph showing that chronic rapamycin treatment did not alter mean paired-pulse ratios. (**H**) Sample traces of mIPSCs. (**I**) Chronic rapamycin treatment significantly increased mIPSC frequency. (**J**) mIPSC cumulative amplitude plot. (**K**) mIPSC inter-event interval histograms showing that a significantly greater proportion of GABAergic events occurred in closer proximity after chronic rapamycin treatment.

Spontaneous synaptic activity was examined in voltage clamp mode using Cs-methanesulfonate as the internal solution. Chronic rapamycin treatment had no effects on sEPSCs mean frequencies, cumulative amplitude and inter-event interval histograms, or average event kinetics. Similarly, there were no differences in mEPSCs. Recordings of sIPSCs were performed in the presence of ionotropic glutamate receptor antagonists. Chronic rapamycin treatment in normal mice did not result in differences in the mean frequency of sIPSCs (7.3 ± 1.2 Hz in vehicle vs. 7.0 ± 1.7 Hz in rapamycin-treated animals) (Figure 7A,B). There were also no differences in the amplitude–frequency, cumulative amplitude and inter-event interval distributions. The kinetics of averaged GABAergic events also were unchanged.

To determine if there were any differences in the AP-independent release of GABA, mIPSCs were recorded after bath applying TTX (Figure 7H). Normal cells from rapamycin-treated animals displayed an increase in mIPSC frequency (2.63 ± 0.36 Hz in vehicle vs. 6.42 ± 1.5 Hz in rapamycin-treated animals, *p* = 0.01) (Figure 7I). Cells from rapamycin-treated animals also displayed a lower percentage decrease in frequency in the presence of TTX compared with cells from vehicle-treated animals (−53 ± 7% in vehicle vs. −28 ± 9% in rapamycin-treated animals, *p* = 0.05). This finding suggests that even though sIPSCs were unchanged with treatment, more GABAergic synaptic events are independent of APs in the rapamycin-treated animals. There was only a slight but not statistically significant effect of rapamycin on the cumulative amplitude probability plot (F_(1,10)_ = 2.844 for treatment effect, *p* = 0.1226) (Figure 7J). The inter-event interval probability histogram demonstrated that mIPSCs occurred closer together temporally in rapamycin-treated animals (F_(1,10)_ = 10.83 for treatment effect and F_(40,400)_ = 9.823 for interaction effect, *p* = 0.0081 and *p* < 0.001 respectively) (Figure 7K).

Chronic rapamycin treatment in the normal cortex also altered the eIPSC input–output functions of CPNs (Figure 7C). This resulted in decreased eIPSC amplitudes (F_(1,13)_ = 6.309 for treatment effect, *p* = 0.026) (Figure 7D) and a trend of shorter decay times compared to those of vehicle-treated controls (F(_1,13)_ = 2.976 for treatment effect, *p* = 0.108) (Figure 7E). To determine if the change in eIPSC amplitudes was due to a change in the Pr, paired-pulse ratios were examined (Figure 7F,G). There were no treatment-specific differences, with PPD present in both groups at 100, 200 and 400 ms (and facilitation at 25 ms).

## 4. Discussion

Using morphological, electrophysiological, and pharmacological methods, we demonstrated the critical role of the mTOR pathway in the regulation of CPN excitability in both normal and diseased brains. Disinhibition of mTOR through conditional Pten deletion in CPNs induced robust changes in basic membrane properties, membrane excitability, and synaptic function. Rapamycin treatment prevented the effects of Pten loss on basic membrane properties, synaptic inhibition, and intrinsic excitability.

### 4.1. mTOR Modulates Cell Soma Size and Basic Membrane Properties

Neurons from Pten^-/-^ animals had higher capacitances and longer time constants than cells from WT animals. The changes in capacitance suggest that cells from the Pten^-/-^ neocortex are larger. Measurements of the cross-sectional somatic area from the DIC images were consistent with the hypothesis that cells from the Pten^-/-^ neocortex were larger. Cells recorded from rapamycin-treated Pten^-/-^ animals demonstrated a prevention of changes in capacitance, input resistance, and time constant compared to vehicle controls, consistent with a reduction in somatic area. Cell areas measured from IR-DIC images were also reduced after rapamycin treatment. This is not surprising, given that previous work showed that rapamycin improves the morphological abnormalities following Pten deletion [20] and is consistent with the known roles of mTORC1 and mTORC2 in cell growth and cytoskeletal function. One potential byproduct of these changes, however, is an effect on cable properties. An increase in input resistance will serve to amplify dendritic currents as they are passively conducted toward the soma [35]. We have to acknowledge that our study has important limitations in that we were not able to differentiate CPNs lacking Pten expression. However, we could speculate that cells with soma sizes larger than 200 µm^2^ (about 50% of CPNs recorded) lost Pten expression, as demonstrated by another study using the same mouse model [31].

### 4.2. mTOR Influences Intrinsic Membrane Excitability

Rapamycin-treated WT and Pten^-/-^ CPNs were more hyperpolarized at rest. There are several possible explanations for the robust hyperpolarization observed in rapamycin-treated neurons. A study demonstrated that long-term exposure to rapamycin resulted in an upregulation of Kir1.1, an inwardly rectifying K^+^ channel [36]. The Kir channels are critical to establishing the RMP [37]. Thus, overexpression of these channels may result in increased K^+^ permeability and a RMP that approaches E_K_. In non-neuronal cells, p70S6K (a major effector of mTORC1) has been shown to be critical in the regulation of Na^+^/K^+^ ATPase expression, suggesting that rapamycin also may affect RMP by changing electrochemical gradients driving leak conductance [38].

All rapamycin conditions (WT and Pten^-/-^) resulted in increases in ΔV. Cells from animals with chronic exposure to rapamycin had a greater ΔV that was attributable to a more hyperpolarized RMP without a change in the AP threshold. In WT CPNs, the threshold for firing was not significantly affected, whereas the RMP was significantly hyperpolarized, which led to a reduction in AP generation. In the rapamycin-treated Pten^-/-^ condition, however, the ΔV was reflective of both a more hyperpolarized RMP and a more depolarized threshold for AP generation. The threshold for AP initiation is thought to be determined by the rate of change in the membrane potential [39]. This is most likely determined by a Na^+^ conductance [39,40,41]. Subthreshold I_Na_ is modulated either by regulating the availability of Na^+^ channels at a given membrane potential or by changing the conductance of I_NaP_, the persistent subthreshold Na^+^ current [41]. While rapamycin has not yet been shown to directly affect neuronal I_Na_ in vitro, it has been shown to change Na^+^ conductances in other cell types [42]. Furthermore, in a model of sudden unexpected death of epileptics (SUDEP) produced by the deletion of nitrogen permease regulator-like 2 (NPRL2), upregulation of Na^+^ channel expression can be prevented by treatment with rapamycin [43].

### 4.3. mTOR Modulates Spontaneous and Evoked GABAergic Signaling

Unlike prior work on dentate hippocampal cells [44], mTOR modulation had little effect on EPSCs but had major effects on IPSCs in the neocortex. With chronic rapamycin treatment, there was an increase in mIPSC frequency, with events in closer temporal proximity. This suggests a change in AP-independent GABAergic transmission not reflected by sIPSC frequency. The increase in mIPSC frequency, along with the reduction in inter-event intervals, suggests an enhanced presynaptic release of GABA independent of APs. The slight leftward shift in the cumulative amplitude plot may represent fewer postsynaptic GABA_A_ receptors [45] or GABA_A_ receptor desensitization.

There was a reduction in sIPSCs and mIPSCs in the Pten^-/-^ neocortex that was prevented by rapamycin treatment. The reduction in mIPSC frequency was not driven by reductions in the AP-dependent release of GABA, which is a particularly interesting finding considering that Pten loss was confined to CPNs. The data suggest that the reduction in GABA_A_ receptor signaling was due primarily to presynaptic changes, since there was a leftward shift in the cumulative inter-event interval, indicating a reduction in the temporal proximity of events.

Rapamycin exposure resulted in reductions in eIPSC amplitudes, whereas the Pten^-/-^ neocortex exhibited large eIPSC amplitudes. This is not explained by a change in release probability, as paired-pulse ratios were unaffected [32]. Previous evidence has suggested that there are multiple pools of vesicular GABA which may contribute to differential evoked and miniature synaptic currents [46]. One explanation for this difference in rapamycin’s effect on mIPSCs and eIPSCs may be due to an increase in GABA vesicle pools allocated for spontaneous release and a reduction in those available for evoked release. Another possible explanation could be a reduction in the number of readily releasable vesicles at the synapse (n), suggesting a change in presynaptic function. It also is possible that mTOR induces a change in postsynaptic GABA_A_ receptors and their sensitivity. This idea is further substantiated by the prolonged eIPSC decay times present in the Pten^-/-^ neocortex and prevented by rapamycin treatment. These changes in eIPSCs are consistent with observations made in Pten-deficient hippocampal and striatal cell cultures [23]. In these studies, increases in mTOR signaling were noted to be secondary to increases in the number of synaptic vesicles available for release and the number of synapses formed. Finally, it also is possible that reduced presynaptic release of GABA in Pten-deficient mice induces homeostatic postsynaptic changes leading to an increased number and sensitivity of GABA_A_ receptors [47].

**Figure 8:**
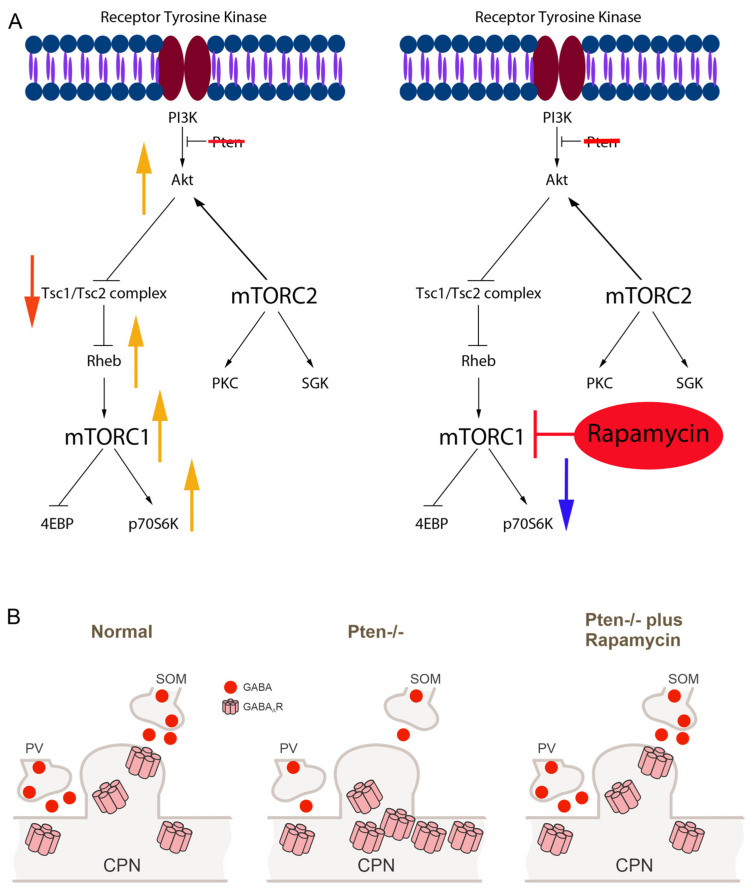
(**A**) Pten is upstream of the mTORC1 and mTORC2 protein complex and negatively regulates their functions. Rapamycin administration acts to inhibit mTORC1. Conditional deletion of Pten from cortical pyramidal neurons (CPNs) increases somatic areas and reduces the frequency of spontaneous IPSCs, while it increases the amplitude of evoked IPSCs (left panel). Chronic rapamycin treatment prevents these alterations by blocking mTORC1 (right panel). (**B**) The mTOR pathway, via autophagy, regulates surface expression of GABA_A_ receptors, which could explain postsynaptic homeostatic changes in Pten-deficient CPNs.

Although the intimate molecular mechanisms of mTOR pathway and GABA_A_ receptor interactions remain unknown, studies have shown possible nodes of interaction (Figure 8B). For example, γ-aminobutyric acid receptor-associated protein-like 2 (GABARAPL2) forms high-molecular-weight species in autophagy-deficient brains, and their sequestration leads to alterations in surface GABA_A_ receptor levels. This may reveal a potential mechanism for the reduced inhibitory inputs observed in neurological disorders with mTOR hyperactivation including epilepsy and autism [48].

## 5. Conclusions and Implications

This series of experiments demonstrated the necessary and sufficient role of mTOR in the regulation of neocortical excitability. Rapamycin treatment induced alterations in basic membrane properties, a reduction in membrane excitability, and changes in GABAergic signaling. The synaptic data suggest that the mTOR pathway within CPNs may play a role in the presynaptic regulation of inhibitory cortical interneurons. This is particularly intriguing in that it represents a non-cell autonomous effect in the Pten-/- mutants, such that affected CPNs influence the AP-independent release of GABA from local circuit interneurons.

Interestingly, rapamycin had an effect in both normal and abnormal neurons, strengthening the notion of bidirectional modulation by mTOR. From a therapeutic perspective, this implies that rapamycin treatment could induce abnormalities in normal cells existing alongside cells with mTOR overactivation. Finally, from a disease perspective, Pten deletion was associated with changes in IPSCs, consistent, primarily, with reduced presynaptic GABA release. This suggests that Pten-related seizures may be due to reductions in functional GABAergic inhibition and might be driven in part by interneuron alterations. These in vitro findings correlate well with the reported seizure susceptibility of animals with reduced Pten signaling [20]. Indeed, preliminary observations in our Pten^-/-^ mice showed that the seizure latency after systemic picrotoxin administration was significantly reduced compared to that in control mice, and that the latency increased in animals that received chronic rapamycin injections. This may have significant implications for the treatment of diseases such as TSC, where mTOR hyperactivation leads to cortical excitation–inhibition imbalance and epilepsy.

The present findings form a physiological foundation for the use of mTOR antagonists to understand and/or treat these diseases. In fact, several studies have shown that rapamycin and everolimus have antiseizure effects in epilepsy models and in human ex vivo slices from TSC patients [49,50,51]. Even though these rapalogs have poor penetration across the blood–brain barrier (which explains the need for chronic administration), small-molecule 1,3,5-triazine derivatives, in particular PQR620, a catalytic mTORC1/mTORC2 inhibitor, rapidly enter the brain, decrease the phosphorylation of S6 ribosomal protein, and increase the seizure threshold at tolerable doses [52].

Our findings could also have important implications for autism, another phenotype of Pten deletion. While most studies have focused on glutamate neurotransmission dysfunction, accumulating evidence points to altered GABA signaling in autism spectrum disorders as well. For example, parvalbumin interneuron numbers are reduced, and deficits or aberrant GABA neurotransmission have been amply demonstrated [53,54,55,56,57]. Our finding that alterations mainly affect GABA signaling in Pten^-/-^ mice gives evidence for the critical role of this signaling pathway in maintaining the excitatory/inhibitory balance.

## Data Availability

The data supporting this article are available from the corresponding author on reasonable request.
